# The Phylogenetic Relationships of Major Lizard Families Using Mitochondrial Genomes and Selection Pressure Analyses in Anguimorpha

**DOI:** 10.3390/ijms25158464

**Published:** 2024-08-02

**Authors:** Lemei Zhan, Yuxin Chen, Jingyi He, Zhiqiang Guo, Lian Wu, Kenneth B. Storey, Jiayong Zhang, Danna Yu

**Affiliations:** 1 College of Life Sciences, Zhejiang Normal University, Jinhua 321004, China; 2 Department of Biology, Carleton University, Ottawa, ON K1S5B6, Canada; 3 Key Laboratory of Wildlife Biotechnology, Conservation and Utilization of Zhejiang Province, Zhejiang Normal University, Jinhua 321004, China

**Keywords:** Squamata, Anguimorpha, phylogenetic relationships, mitogenomes, positive selection, limbless lizards

## Abstract

Anguimorpha, within the order Squamata, represents a group with distinct morphological and behavioral characteristics in different ecological niches among lizards. Within Anguimorpha, there is a group characterized by limb loss, occupying lower ecological niches, concentrated within the subfamily Anguinae. Lizards with limbs and those without exhibit distinct locomotor abilities when adapting to their habitats, which in turn necessitate varying degrees of energy expenditure. Mitochondria, known as the metabolic powerhouses of cells, play a crucial role in providing approximately 95% of an organism’s energy. Functionally, mitogenomes (mitochondrial genomes) can serve as a valuable tool for investigating potential adaptive evolutionary selection behind limb loss in reptiles. Due to the variation of mitogenome structures among each species, as well as its simple genetic structure, maternal inheritance, and high evolutionary rate, the mitogenome is increasingly utilized to reconstruct phylogenetic relationships of squamate animals. In this study, we sequenced the mitogenomes of two species within Anguimorpha as well as the mitogenomes of two species in Gekkota and four species in Scincoidea. We compared these data with the mitogenome content and evolutionary history of related species. Within Anguimorpha, between the mitogenomes of limbless and limbed lizards, a branch-site model analysis supported the presence of 10 positively selected sites: Cytb protein (at sites 183 and 187), ND2 protein (at sites 90, 155, and 198), ND3 protein (at site 21), ND5 protein (at sites 12 and 267), and ND6 protein (at sites 72 and 119). These findings suggested that positive selection of mitogenome in limbless lizards may be associated with the energy requirements for their locomotion. Additionally, we acquired data from 205 mitogenomes from the NCBI database. Bayesian inference (BI) and Maximum Likelihood (ML) trees were constructed using the 13 mitochondrial protein-coding genes (PCGs) and two rRNAs (12S rRNA and 16S rRNA) from 213 mitogenomes. Our phylogenetic tree and the divergence time estimates for Squamata based on mitogenome data are consistent with results from previous studies. Gekkota was placed at the root of Squamata in both BI and ML trees. However, within the Toxicofera clade, due to long-branch attraction, Anguimorpha and (Pleurodonta + (Serpentes + Acrodonta)) were closely related groupings, which might indicate errors and also demonstrate that mitogenome-based phylogenetic trees may not effectively resolve long-branch attraction issues. Additionally, we reviewed the origin and diversification of Squamata throughout the Mesozoic era, suggesting that Squamata originated in the Late Triassic (206.05 Mya), with the diversification of various superfamilies occurring during the Cretaceous period. Future improvements in constructing squamate phylogenetic relationships using mitogenomes will rely on identifying snake and acrodont species with slower evolutionary rates, ensuring comprehensive taxonomic coverage of squamate diversity, and increasing the number of genes analyzed.

## 1. Introduction

Lizards are renowned for their remarkable diversity within the reptilian order Squamata. The Reptile Database (http://www.reptile-database.org/, accessed on 9 March 2024) documents an impressive 7415 species of lizards, which is the highest compared to snakes (4073) and amphisbaenians (202), distributed among 36 families [1]. These adaptable creatures can be found in a wide range of terrestrial habitats worldwide, with the exception of polar regions. The major lineages of lizards have exhibited significant specialization in terms of their physical traits, behaviours, and ecological roles [2,3]. However, in the squamate reptiles, there is currently insufficient evidence to conclusively establish the true relationship between lizards, amphisbaenians, and snakes, as well as the high-level classification of lizards. Since the groundbreaking classification study based on both morphological and fossil evidence by Camp et al. [4], there has been an ongoing controversy regarding the phylogenetic relationships within the order Squamata. A robust phylogeny of squamate reptiles remains elusive, with different evidence pointing in different directions. On the basis of morphological and fossil data, Iguania was considered to be the most morphologically conserved [5,6,7,8] (Figure S1). However, in morphological data, some taxa with many convergent trait states may be misplaced due to homoplasy, such as Scincoidea, a paraphyletic group consisting of some legless skinks, Dibamidae + Amphisbaenia, *Anniella* (an anguimorph), and Serpentes [9]. Molecular studies indicated that the Iguania lineage, which used tongue protrusion to capture food, was a highly derived group of lizards and did not represent the primitive evolutionary state of squamate reptiles [10]. Use of molecular data also indicated that limbless dibamids and/or geckos were the first diverging branches of squamate reptiles. It is worth noting that Simões et al. [11], based on morphological and molecular data, had reached a consensus on the evolution of Squamata, suggesting that geckos were the first to diverge within Squamata, rather than Iguania (Figure S1). Furthermore, studies among molecular hypotheses show consistency in reflecting identical uncertain regions in the tree, such as monophyly issues within the Toxicofera clade and the placement of dibamids.

In recent years, mitogenomes have been considered a promising molecular marker in systematic biology and have been widely utilized in the analysis of phylogenetic relationships. This is attributed to the fact that mitogenomes (composed of 13 PCGs, 22 tRNA genes, 2 rRNA genes, and a noncoding control region of varying lengths) are inherited extranuclear [12,13]. They possess several advantageous characteristics, including a relatively small molecular size, a simple structure, a faster evolutionary rate, and variable rates of evolution within and among populations [14,15,16]. Meanwhile, mitochondria are essential organelles responsible for energy production, and their 13 PCGs are closely linked to cellular metabolism [17,18,19]. There has been a growing trend in research to combine the study of adaptive evolution in species with the unique features of mitochondria, highlighting their significance in shaping evolutionary processes. In fact, adaptive selection was considered a primary factor influencing mitochondrial evolution or changes in codon usage. Evidence suggested that genes involved in energy regulation within mitochondria had shown direct responses to selection pressure. In reptile groups, positive selection of mitogenomes has been found in Tibetan sand lizards living in high-altitude areas, compared to groups adapted to low-altitude environments [20]. Similarly, in amphibians such as *Hyla* and *Dryophytes*, the mitogenomes underwent positive selection, possibly due to the need for more energy to adapt to low-temperature environments [21]. It is worth noting that the mitogenomes of the limbless skink *Isopachys gyldenstolpei* had undergone positive selection [22]. This suggested that positive selection pressures on the mitogenome may have played an important role in the dispersal of limbless lizards and their adaptation to habitat environments.

However, mitogenomes of squamate reptiles are not as well-represented as in other species. In previous studies using mitogenomes to resolve complex evolutionary relationships within squamate reptiles, only the mitogenomes of a few species have been utilized. In this study, we newly sequenced mitogenomes of eight squamate species (*Pseudopus apodus*, *Dopasia gracilis*, *Cyclodomorphus gerrardii*, *Chalcides ocellatus*, *Tiliqua gigas gigas*, *Plestiodon quadrilineatus*, *Gekko chinensis*, and *Gekko japonicus*), including four previously unreported ones. We compared these data with mitogenome sequences and evolutionary histories of related species. In the obtained mitogenome sequences of eight species, including two limbless lizards (*P. apodus* and *D. gracilis*), they belong to Anguimorpha. Although relatively fewer in number (about 250 species) compared to other groups of squamate reptiles, Anguimorpha is a diverse group. Within different habitats and ecological niches, there exist variations in limb characteristics, ecological morphology, physiological ecology, and evolution. For instance, limbless groups (Anguinae and Anniellinae), fully developed terrestrial species (Gerrhonotinae, Xenosauridae, and Helodermatidae), and even semi-aquatic lizards (*Shinisaurus crocodilurus* and *Lanthanotus borneensis*). Therefore, Anguimorpha serves as a good model to test whether the mitogenome of limbless lizards undergoes selection pressure during adaptation to habitats.

Anguimorpha are currently classified into seven families including Helodermatidae, Shinisauridae, Varanidae, Diploglossidae, Xenosauridae, Lanthanotidae, and Anguidae; however, achieving broad consensus on their interrelationships remains challenging. They possess rich fossil records dating back as far as 130 million years ago. During the Paleogene, extinct relatives of varanids also existed, such as palaeovaranids. Palaeovaranidae and *Palaeovaranus* had a limited geographical presence, occurring from the early Eocene to the early Oligocene in Europe [23]. The micro-CT scan of the dentary from Dielsdorf verifies the occurrence of plicidentine in *Palaeovaranus* [24], a characteristic that has been previously proposed for that genus [23]. It is worth noting that plicidentine is also found in various vertebrate groups, including varanids [23,25,26,27]. In the Anguidae family, alongside the three extant lineages (Anniellinae, Anguinae, and Gerrhonotinae), the extinct Glyptosaurinae was present during the Cretaceous and Paleogene periods [28,29]. From the existing groups, in terms of phylogenetic relationships, although Anguinae and Anniellinae are both limbless, Anguinae and Gerrhonotinae are sister branches, rather than Anniellinae [30]. Of these two limbless groups, it is notable that Anguinae have a widespread distribution, covering North America, Indonesia, and Europe, as well as parts of Asia and North Africa [31,32]. During the Paleocene and (especially) the Eocene, the highest temperatures of the Cenozoic Era are observed [33,34]. A plethora of squamates [35,36,37,38], including anguids [37], are known to have originated from this period. Most of these species’ close relatives inhabited warm regions [37]. As the Eocene gave way to the Oligocene, a cooling trend ensued, causing a retreat of tropical elements from the north [39,40,41,42], leading to the fragmentation of forests and the emergence of grassland-dominated habitats [43]. The subfamily Anguinae, characterized by a monophyletic group of elongated limbless grass-swimming ecomorphs, successfully expanded from the New World to other continents, achieving wide dispersal and demonstrating cryptic diversity in many lineages [44]. *Anguis* and *Pseudopus* formed a sister clade in Europe, despite their dissimilar morphologies. The genus *Pseudopus* is the largest and most robust taxon within the subfamily Anguinae, first appearing in central Europe approximately 18.5 million years ago [45,46]. *Dopasia* belonged to the Asian lineage and formed a continental clade with *Ophisaurus* from the North American lineage [44]. Augé [47] named all extinct and extant species living in the Cenozoic of Eurasia and North Africa as *Dopasia*, where they were previously known as *Ophisaurus*. Phylogenetic relationships suggested that all species living in Southeast Asia, North America, and North Africa *Ophisaurus* requires more caution [48]. There were numerous *Ophisaurus* fossils in Europe from the Paleogene and Neogene periods [49,50,51,52,53], most of which were described based on parietals. However, Klembara et al. [54] pointed out that *Ophisaurus* (including *Dopasia*) could be distinguished from *Anguis* and *Pseudopus* by the morphology of the dentaries. The extant species of *Ophisaurus* of Southeast Asia are considered descendants of the European Neogene species of *Ophisaurus*. Extant Southeast Asian *Ophisaurus* are considered descendants of European Neogene *Ophisaurus*. It was most likely that *Ophisaurus* migrated from East Asia to North America via the Bering Strait, a notion supported in the late 1970s by the dorsal vertebra evidence in the late Miocene of Canada [55]. The presence of fossils strongly suggested that Western and Central Europe were the origin of *Ophisaurus* [51]. Fossil anguine lizard specimens from several Turkish localities also provided important information about the dispersal routes of anguines from Europe to Asia [56]. Due to limited molecular data and the nature of their natural habitat, there was still much to be explored regarding the phylogenetic placement and species diversity within Anguinae.

The process of movement or range expansion in Anguinae is energy-consuming, with different movement patterns requiring varying levels of energy expenditure. The loss of limbs or injuries to locomotion-related appendages may result in additional energy costs [57]. Many pieces of evidence suggested that the loss of limbs and elongation of the body in lizards were related to adaptation to different environments [58,59,60]. As a low ecological niche, Anguinae, having a snake-like body shape, enables faster undulatory movement [61]. Therefore, we hypothesize that within the Anguimorpha superfamily, mitogenomes of limbless lizards may have undergone positive selection compared to their fully limbed counterparts. Here, we utilized these data in conjunction with published data on the NCBI website (https://www.ncbi.nlm.nih.gov, accessed on 28 June 2024), placing species within the phylogeny and testing the validity of limb-loss characteristics in the Anguimorpha superfamily of limbless lizards. Furthermore, by considering the unique morphological characteristics and divergence times of Anguinae, it is speculated whether limblessness in Anguinae represents an adaptation to fragmented habitats, with the aim of expanding distribution.

## 2. Results

### 2.1. Basic Features of Mitogenomes

**Figure 1:**
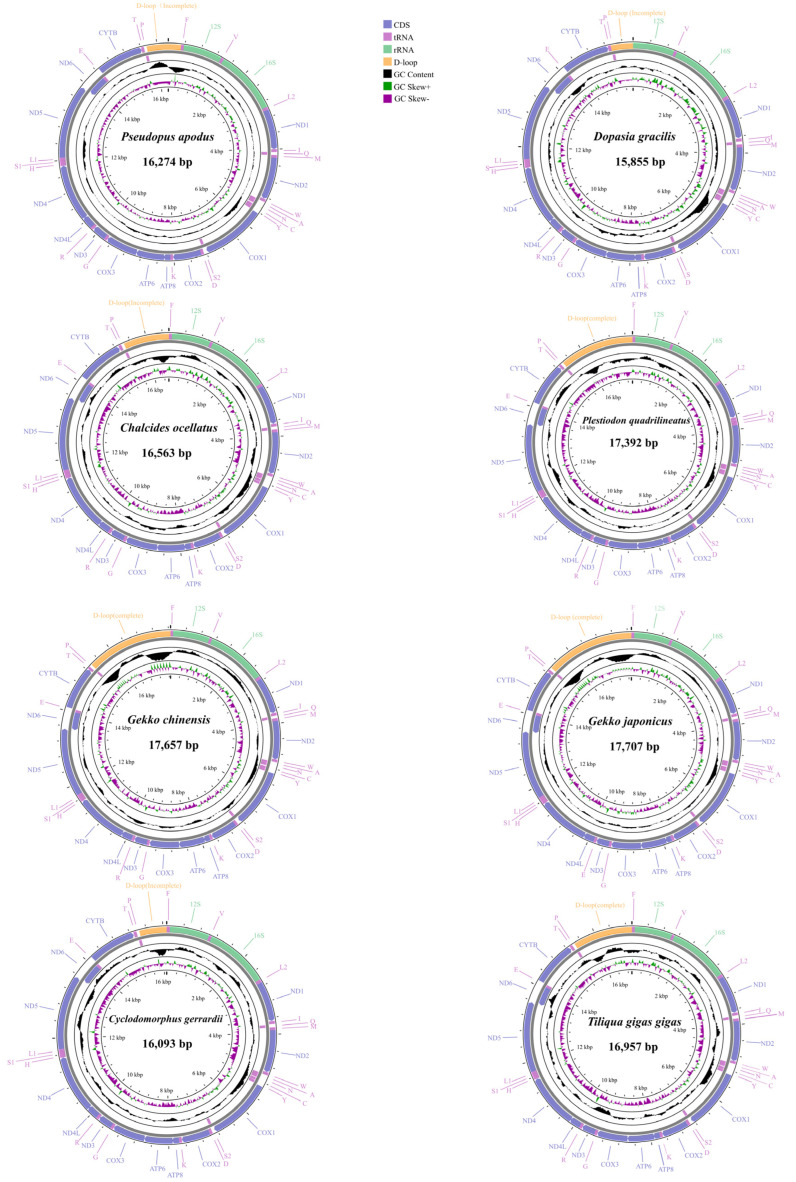
Mitogenome map of eight species in this study. The outermost two circles depict the gene map (PCGs, rRNAs, tRNAs, and D-loop region) and genes; the outer circle is encoded by the majority strand, the second circle is encoded by the minority strand, and the tRNAs are all denoted by abbreviations. The black circle represents GC content, whereas the circles are composed of green, and violet represent GC skew.

In the present study, we obtained eight mitogenomes, including four complete mt genomes (*T. gigas gigas*: 16,957 bp, *P. quadrilineatus*: 17,391 bp, *G. chinensis*: 17,659 bp, *G. japonicus*: 17,707 bp) and four nearly complete mitogenomes, ranging in size from 15,855 bp for *D. gracilis* to 16,563 bp for *Ch. ocellatus* (Figure 1, Table 1). Within limbless lizards, the complete mitogenome of *P. apodus* was found to have a composition of 30.7% A, 24.5% T, 14.7% G, and 30.2% C. Similarly, *D. gracilis* exhibited a mitogenome composition of 30.5% A, 23.8% T, 15% G, and 30.7% C (Table S1). In conformity with the observed patterns in other limbed lizards, the mitogenomes of *P. apodus* and *D. gracilis* displayed a pronounced AT bias. The respective AT content, CG skew, and AT skew values for each species are tabulated in Table 1, reflecting these genomic characteristics. These findings manifested a clear preference for A + T in the complete mitogenome sequences of lizard species (Table 1).

In the mitogenome of eight lizard species, varying lengths of gene spacers and overlaps were observed (Table S2). The number of gene spacer regions ranged from 9 to 13, with gene spacer lengths ranging from 1 to 18 bp across all eight lizard species. The longest spacer regions appeared at five positions, *COI*-*trnS*, *ND1*-*trnL*, *trnI-trnQ*, *trnN-trnC*, and *Cytb*-*trnT*. Among these species, two gecko species had the longest overlaps at 15 bp and 16 bp, both occurring between *ND5* and *ND6*. The remaining six species had the longest overlaps of 10 bp, found between the *ATP8* and *ATP6* genes.

The 13 PCGs composition of eight lizards is shown in Table 1. The *Ch. ocellatus* and *P. apodus* mitogenomes exhibited a relatively long length for their 13 PCGs, measuring 11,388 base pairs. In contrast, *G. chinensis* had the shortest PCGs length, only 11,298 base pairs. Among the 13 PCGs in eight species, only *ND6* was located on the light strand, with the remaining 12 PCGs located on the heavy strand (Figure 1 and Table S2). Assessment of PCG sequences revealed a clear bias toward AT, with positive AT skew and negative GC skew for the genes situated on the heavy chain, whereas the genes on the light chain displayed negative AT skew and positive GC skew (Table 1 and Table S1).

In terms of initiation codon usage (Table S2), *COI*, *ND2*, and *ND6* exhibited distinct patterns among the genes analyzed. Across all other genes, ATG served as the initiation codon. For *COI*, all species employed GTG as the initiation codon. Among the two species in Gekkonidae, *G. chinensis* and *G. japonicus*, the *ND2* gene started with ATT. Additionally, only the *ND6* gene in *P. apodus* started with GTG, while in all other species, ATG was utilized as the initiation codon. Among these 13 PCGs, most genes used complete termination codon (TAA and TAG), with AGA being the most frequently used termination codon in the *COI* gene of lizard species. A few genes used incomplete termination codons (T and TA), particularly noticeable in *ND2*, *ND3*, *ND4*, *COII*, and *COIII* (Table S2). These T/TA termination codons were converted into intact TAA stop codons via post-transcriptional polyadenylation.

**Figure 2:**
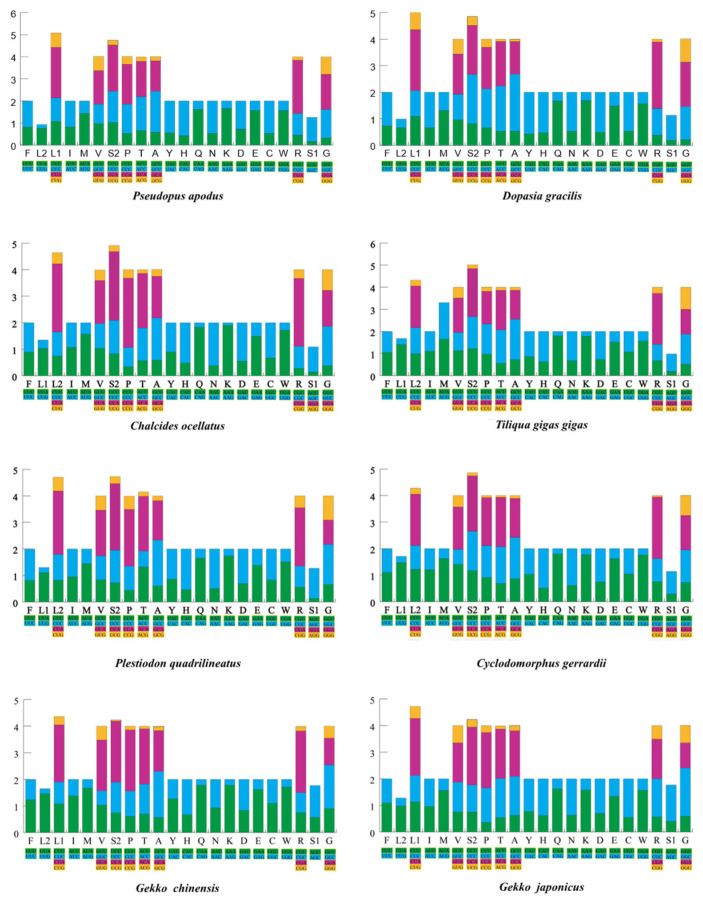
The relative synonymous codon usage (RSCU) of the mitogenomes of eight species in this study. The X-axis displays all the utilized codons, including various combinations of synonymous codons, where each codon is depicted with a distinct color. The Y-axis presents the corresponding RSCU values in a list format. Different codons are shown in the different colors.

In the analysis of the RSCU (Figure 2 and Figure S2) and amino acid composition of the 13 PCGs (Table S3), *G. chinensis* and *G. japonicus* from the Gekkonidae family had a relatively lower total number of codons, excluding termination codons, compared to other species (3781–3788), specifically 3756 and 3762, respectively. Among the 60 amino acid codons in *P. apodus*, 29 codons were used more frequently (RSCU > 1), and 31 codons were used less frequently (RSCU < 1). Similarly, *D. gracilis* used 27 codons more frequently (RSCU > 1) and 33 codons less frequently (RSCU < 1). Both species demonstrated a high frequency of the CGA codon encoding arginine (Arg), with frequencies of 2.28 and 2.29 for *P. apodus* and *D. gracilis*, respectively. It is noteworthy that the CGA codon, as the most frequently used codon, also appeared in *T. gigas gigas*, *Cy. Gerrardii,* and *G. chinensis*.

Except for *D. gracilis*, the remaining seven species of lizards had tRNA lengths ranging from 1533 to 1546 bp. Seven tRNAs were located on the negative strand (trnQ, trnA, trnN, trnC, trnY, and trnS), while the remaining tRNAs were on the positive strand (Table S2). Most tRNAs could be folded into canonical cloverleaf structures. The control region was located between trnP and trnF. The length of 16S rRNA ranged from 1533 bp (*P. quadrilineatus*) to 1570 bp (*G. chinensis*), and that of 12S rRNA ranged from 943 bp (*P. apodus*) to 965 bp (*G. chinensis*) (Table S2). Both rRNAs exhibited a negative AT-skew, a positive GC-skew, and high AT content (from 53.5% to 58.4%) (Table S1). Based on these results, the mitogenome structure of the two limbless lizards was not significantly different from that of limbed lizards.

### 2.2. Phylogenetic Relationships

**Figure 3:**
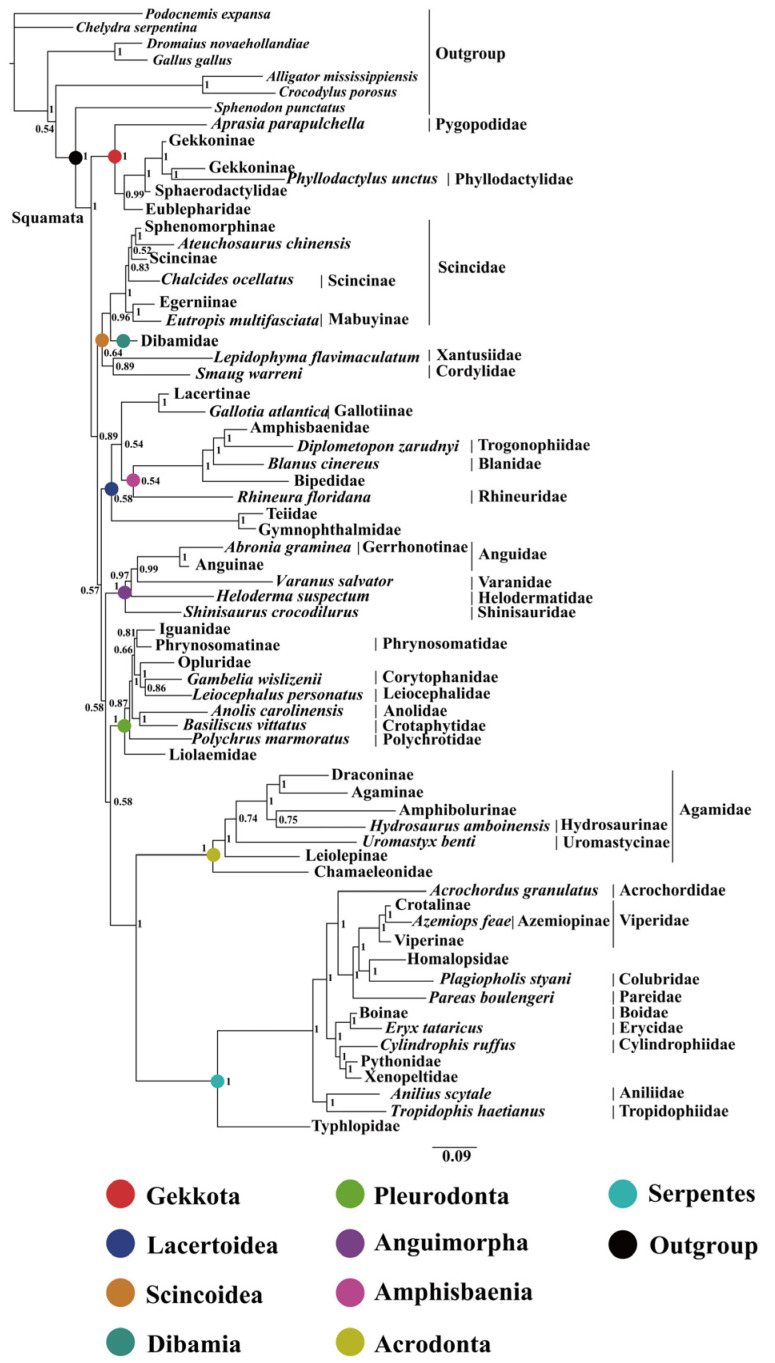
BI tree constructed with 13 protein-coding genes (the first and the second codons) and two rRNAs (12S rRNA and 16S rRNA) based on 213 datasets. Summary of the relationships between higher-level branches of squamate reptiles estimated in this study, with numbers at the nodes indicating bootstrap support values (the full tree is presented in Figure 4, Figure 5 and Figure 6). The same color displayed on both sides of the figure represents the same clade in the phylogeny.

**Figure 4:**
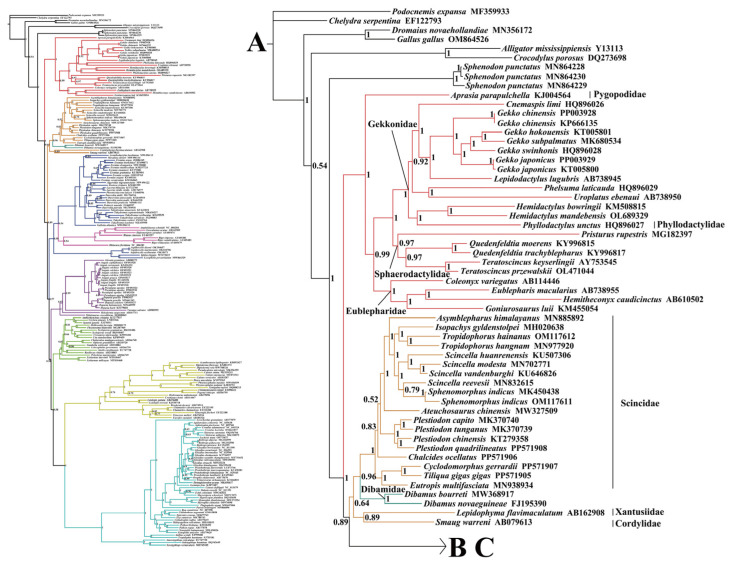
The left side is the skeleton version of 213 mitogenomes, and the right side shows an enlarged view of the corresponding branch on the left side, which is distinguished by different colors. Species-level squamate phylogeny Part A. To illustrate clearly, the complete species-level tree of Squamata in BI tree was divided into three parts: A, B, and C. For Part B and C, please see the next legend.

**Figure 5:**
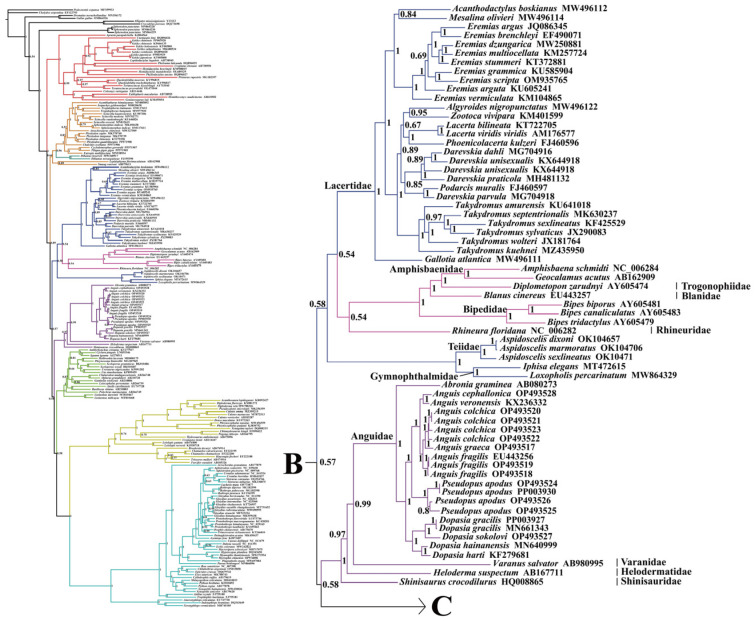
Species-level squamate phylogeny Part B. For Part C, please see the next legend.

**Figure 6:**
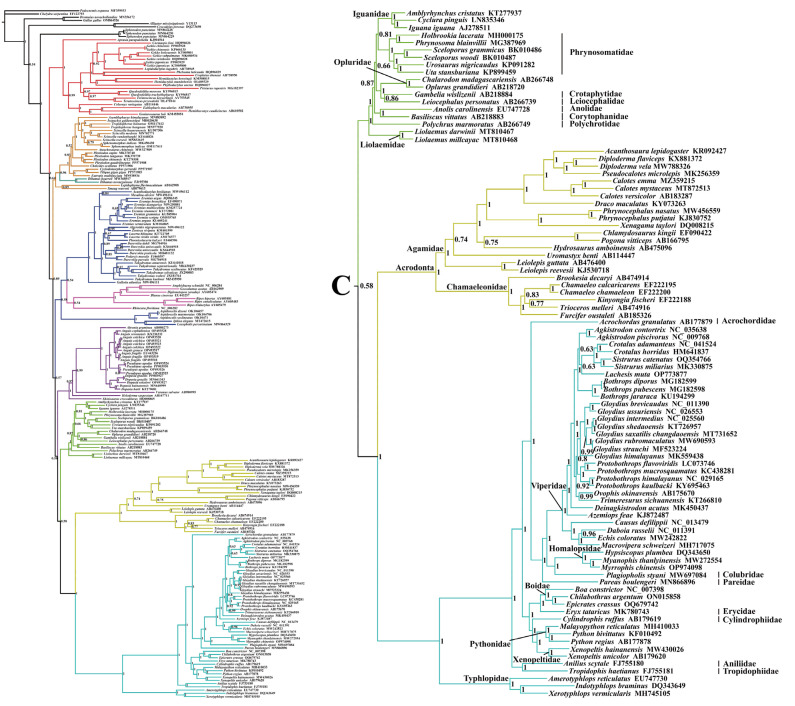
Species-level squamate phylogeny Part C.

This study utilized a dataset comprising nucleotide sequences of 13 PCGs (the first and the second codons) and two rRNAs (12S rRNA and 16S rRNA) extracted from 213 mitogenomes. The phylogenetic tree constructed using BI and ML showed a similar topology with a slight difference (Figure 3, Figure 4, Figure 5 and Figure 6 and Figures S2–S4). The results indicated the following: Both trees recovered the monophyly of Dibamia, Gekkota, Anguimorpha, Amphisbaenia, and Serpentes. Gekkota was placed at the root of the Squamata tree, being the earliest diverging lineage among Squamata. Dibamia and Scincidae formed a clade, which diverged immediately after Gekkota. Rhineuridae in Amphisbaenia first diverged as a sister group to the rest of the amphisbaenians. Amphisbaenia was classified with Lacertidae, and then with Gymnophthalmidae + Teiidae, consistent with the majority of research findings. We recovered the monophyly of Toxicofera, but not that of Iguania. Rather, Serpentes clustered first with Acrodonta and then with Pleurodonta to form a sister branch, with Anguimorpha located at a relatively distant position.

Within Anguimorpha, both the BI and ML analyses produced consistent results (Figure 3, Figure 4, Figure 5 and Figure 6 and Figures S2–S4). Anguidae was more closely related to Varanidae. Anguidae + Varanidae first clustered with Helodermatidae and then formed a sister group with Shinisauridae. Within Anguidae, only one species, *Abronia graminea*, possessed limbs, whereas the remaining species were limbless. In the limbless clade, *Pseudopus*, which included *P. apodus*, formed a clade with *Anguis*. This clade clustered with *Dopasia,* including *D. gracilis*, forming the topology of (*Dopasia* + (*Pseudopus* + *Anguis*)) (PP = 1, BP = 100).

Within Serpentes, except for Leptotyphlopidae, Gerrhopilidae, Xenotyphlopidae, and Anomalepididae, Typhlopidae is the sister group to the remaining snakes. And we restore the monophyly of the Constrictores. Constrictores is considered a valid name at the supra-familial level (order-group name). From the point of view of hierarchy, Constrictores is ranked below the level of Alethinophidia and above the level of the superfamilies Booidea and Pythonoidea [62]. Within Constrictores, the topology formed was ((Boidae + Erycidae) + (Cylindrophiidae + (Pythonidae + Xenopeltidae))). Within the advanced snakes (Caenophidia), here we got a topology of (Acrochordidae + (Pareatidae + (Viperidae + (Homalopsidae + Colubridae)))). In our analysis, Tropidophiidae and Aniliidae appeared as sister groups, therefore supporting the concept of Amerophidia [63,64]. In ML tree, we recovered turtles closer to Archosauromorpha, supporting the concept of Archelosauria (Figure S2), which is also supported by most molecular studies [65].

### 2.3. Divergence Time Estimation

**Figure 7:**
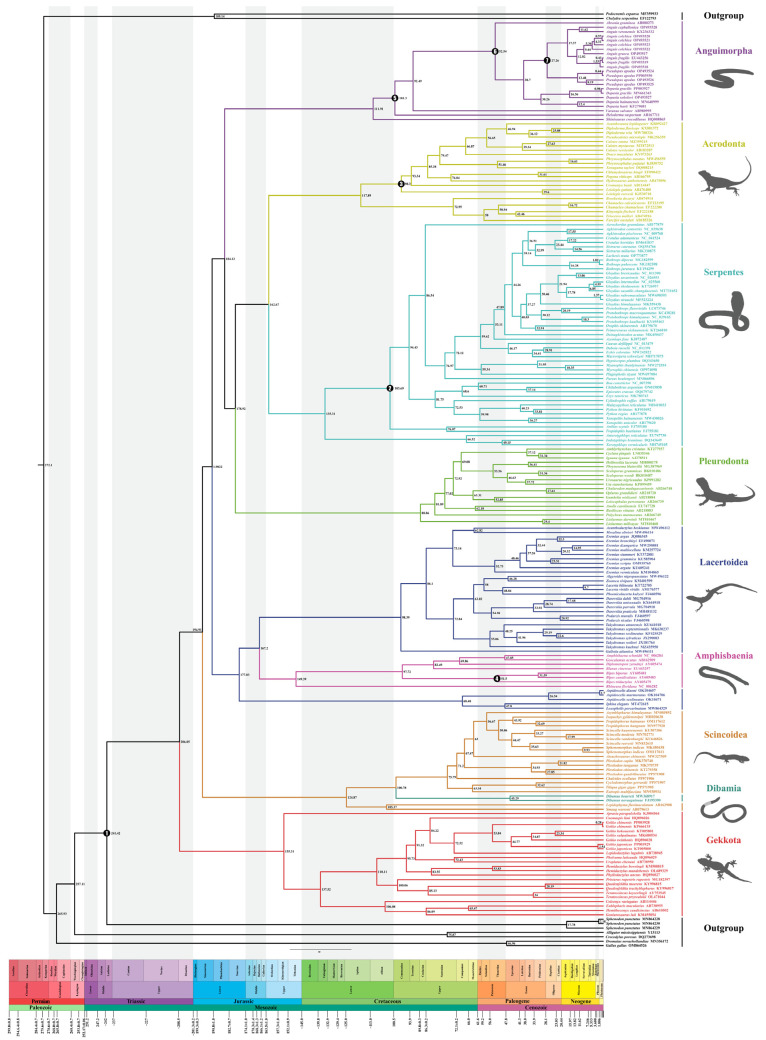
Evolutionary timescale within Squamata based on phylogenetic analyses. Median-divergence times are provided above every node, and the seven fossil calibration points used are marked in the figure. The scale data are from million years ago (Mya), and the geological timescale is shown at the bottom of the chronogram.

Based on the BI tree seen in Figure 3, analysis to estimate the divergence times of 213 samples utilized seven fossil calibration points along the lizard clade’s backbone and one calibration point for Rhynchocephalia–Squamata split. For the crown-group, we recovered Squamata dating back to 206.05 Mya (182.08~229.55) (Figure 7 and Table 2). A significant number of extant clades began to diversify in the Cretaceous period: Anguimorpha (111.91 Mya), Pleurodonta (88.86 Mya), Acrodonta (117.89 Mya), Serpentes (135.31 Mya), Scincidae (75.79 Mya), and Lacertidae (98.39 Mya) (Figure 7 and Table 2). Whereas geckos located at the base of Squamata began to diversify approximately at 155.31 Mya, in the late Jurassic period.

Anguimorpha and (Pleurodonta + (Serpentes + Acrodonta)) were closely related groupings, sharing a common ancestor estimated to have emerged approximately 184.12 Mya (95% HPD: 160.76~206.52 Mya). Additionally, we suggested that limbless lizards originated from a common ancestor approximately 38.70 Mya (95% HPD: 31.18~46.33 Mya) during the Paleogene era. The most significant lineages appeared during the Oligocene [51], with substantial divergence at the species level occurring in the Miocene.

Within the scope of this study, the divergence of *P. apodus*, a species within Anguinae, took place approximately 12.48 Mya (95% HPD: 7.16~18.07 Mya) in the Miocene period. Notably, this divergence event occurred 18 million years after the divergence of *Dopasia*. Simultaneously, *Dopasia* initiated diversification prior to the divergence of the common ancestor of *Pseudopus* and *Anguis.*

### 2.4. Detecting Selective Pressure Within Anguimorpha

Different topological structures of trees can have an impact on the results for selection pressure, whereas the topological structures in the BI tree and ML tree were consistent within Anguimorpha. Based on the results obtained from the BI tree, the branch-site model applied an LRT to compare Model A with Model A null, yielding *p* < 0.05. This significant result indicated that Model A outperformed the Model A null, providing evidence for the presence of positive selection. Specifically, 10 potential sites (1151, 1216, 1259, 1688, 2249, 2504, 3138, 3187, 3464, and 3468) exhibiting positive selection were identified with BEB > 0.95 (Table 3 and Table S4). These sites were distributed among five genes (Table 4), namely, *Cyt b* (two sites), *ND2* (three sites), *ND3* (one site), *ND5* (two sites), and *ND6* (two sites). Furthermore, it is worth noting that mitochondrial complex I was the main protein under selective pressure (Figure S5 and Table 4). Research conducted on the unique characteristics of the positively selected sites within the Anguimorpha branch revealed that nine sites were located in the transmembrane domain of the protein. Similarly, based on the clade model, with limbless lizards in Anguimorpha as the foreground branch and limbed lizards as the background branch, the *p* value for M2a_rel vs. CmC was calculated to be 0.00023 (Table S5). This significant difference between the two hypotheses validated the alternative hypothesis and rejected the null hypothesis, indicating that the foreground clade of limbless lizards in Anguimorpha was subject to positive selection. The evidence from the two models indicated that specific amino acid sites within the sister evolutionary branches composed of *Dopasia*, *Pseudopus*, and *Anguis* may have undergone positive selection in Anguimorpha.

## 3. Discussion

### 3.1. Comparison of Phylogenetic Relationships in Squamates Based on the Mitogenome

Based on the construction of ML and BI trees using 13 PCGs (the first and the second codons) and two rRNAs, the findings of this study were in congruence with earlier research that employed mitogenome data, with the overall topological structure largely maintained, albeit with some branches appearing in a different position. Additionally, the MrBayes method proved to be more effective in resolving the phylogenetic relationships within Squamata based on the mitogenome.

Different topologies based on the mitogenomes could be due to variations in the methods used, the number and selection of taxa, as well as the choices made regarding the number of selected genes. Townsend et al. [66] used only 1175 parsimony-informative sites from 72 mitogenomes to construct the squamate tree. They found that Xantusiidae and Cordylidae clustered together as sister taxa within remaining squamates. Meanwhile, Dibamia and Scincidae formed a sister group. Within the Toxicofera clade, Acrodonta and Serpentes first clustered together, then joined with the Anguimorpha, showing a more distant relationship with Pleurodonta. Additionally, this study did not support the monophyly of the Lacertoidea superfamily.

Böhme et al. [67], used 26 squamate species and six outgroups to construct BI and ML trees based on 13 PCGs. They found that geckos diverged first. However, the monophyly of other clades was not well resolved. The study did not include *Sphenodon punctatus*, considered an appropriate outgroup for Squamata. Albert et al. [68], similar to Böhme in sampling size, used 27 squamate species and nine outgroups to construct trees based on amino acid sequences of 13 PCGs. The resulting topology primarily consisted of two major clades: the first clade comprised of Acrodonta and Serpentes, while the remaining topology included Gekkota and (Amphisbaenia + (Anguimorpha + (Pleurodonta + (Lacertoidea + Scincoidea)))) branches. Incomplete sampling of complete mitogenome within Squamata may have contributed to discrepancies with other molecular studies. The phylogenetic uncertainties underscore the need for more comprehensive mitogenome data in systematic studies.

In the studies by Kumazawa et al. [69] and Okajima and Kumazawa [70], sequences from Dibamia and Serpentes were not included. The resulting topologies were similar: Gekkota diverged first, followed closely by the Scincoidea superfamily. The Lacertoidea superfamily included Amphisbaenia nested within (Iguania + Anguimorpha). In this study, except for the positions of Dibamia and Serpentes, the placements of other clades are consistent with those in these two studies. In squamate phylogenies encompassing comprehensive sampling of squamate taxa (lizards, snakes, amphisbaenians) based on mitogenomes, relationships between Acrodonta and Serpentes, as well as the position of Dibamia, are often contentious. With the development of molecular techniques, any hypotheses suggesting a split within the Acrodonta - Serpentes clade or a closer relationship between Dibamia and Scincidae would be strongly rejected. However, as suggested by other studies, the clustering of Acrodonta and Serpentes may be associated with long-branch attraction [66,67,71]. It is well established that snake lineages exhibit rapid rates of both morphological and molecular evolution, with morphological evolution rates actually much faster than molecular evolution rates [72]. To mitigate this effect, it may be possible to incorporate slower-evolving snakes and Acrodonta species into phylogenetic analyses by identifying those with slower mitochondrial evolution rates. 

From the above discussion, the position of Dibamia may be influenced by the taxonomic sampling and methods used. In this study, utilizing 213 mitogenome sequences (including outgroups), as well as in the studies by Townsend et al. [66], the position of Dibamia was surprising, as it clustered with species from Scincidae, thus disrupting the monophyly of the Scincoidea superfamily. Dibamia represented a very short internode subtending a very long branch [73]. Therefore, it presented challenges for resolution, both morphologically and molecularly. At the same time, phylogenetic relationships based on mtDNA were greatly influenced by sampling within the squamate lineage, and adding mitogenome of species in the Dibamida superfamily will be beneficial for future analyses.

The issue of determining the earliest divergent group within Squamata has long captivated the attention of both morphologists and molecular biologists. Gekkota, positioned as the sister clade to remaining squamates, based on mitogenome, appears stable across studies, strongly rejecting conclusions drawn from morphological studies that categorized squamates into Iguania and Scleroglossa based on differences in tongue structure and feeding habits [9]. In this aspect, and with the positioning of amphisbaenians as a sister group to Lacertidae, molecular data appear to achieve good consistency despite mitochondrial issues such as potential problems like introgression and incomplete phylogenetic classification.

These findings suggest that the use of mitogenomes is not suitable for resolving the long-branch attraction problem, which remains the greatest obstacle in resolving squamate phylogenetics. The major differences in the molecular phylogenetics of Squamata may be attributed to the use of different outgroups, coverage of taxonomic units, or datasets of genetic information. Further exploration is necessary in the future, including the incorporation of more samples and different research methods, ensuring comprehensive taxon coverage of squamate diversity, and incorporating more nuclear genes with suitable evolutionary rates [68]. Currently, studies also implement genome-wide datasets, yet unstable clades persist. Therefore, we should strive to improve models and methods to collect and analyze these datasets, finding the most suitable approaches for analyzing squamate trees.

### 3.2. Analysis of the Phylogenetic Relationships within Anguimorpha

Since Saint et al. [74], Townsend et al. [66], and Vidal and Hedges [75] have demonstrated that snakes, anguimorphs, and iguanians share a more recent common ancestor, excluding other squamates, this branch is collectively referred to as Toxicofera [75]. Moreover, the approximate divergence time of anguimorphs and iguanians is well-documented. However, the relationships between families within the superfamily Anguimorpha continue to be debated. The findings presented by Douglas et al. [76], using the maximum parsimony (MP) tree, demonstrated that Anguidae, Shinisauridae, and/or Xenosauridae were phylogenetically closer to Varanidae than to Helodermatidae. This outcome has garnered support from several scholars [66,77,78,79,80]. However, a study by Douglas et al. [76] using the BI tree of ornithine decarboxylase (OD) data suggested that Helodermatidae had closer relationships to the clade of (Anguidae + Shinisauridae) or Shinisauridae than to Varanidae. In Pyron et al.’s [81] analysis, Xenosauridae was placed in a sister position to Helodermatidae + Anguidae, while in Squamate trees of Zheng et al. [30], Wiens et al. [82], and Burbrink et al. [64], Helodermatidae was the sister lineage of Anguidae + Xenosauridae. Shinisauridae was phylogenetically closer to Varanidae. When morphological and a small amount of molecular data were used, Helodermatidae and Varanidae were more closely related [11]. We found that the relationships among families within the superfamily Anguimorpha, constructed using mitogenomes, are stable, possibly because there is limited mitogenome data available for taxa in this clade, with only one species represented outside of Anguidae. In this present study, we found that Varanidae formed a cluster with Anguidae, successively forming a clade with Helodermatidae and Shinisauridae. These divergent results could potentially be attributed to the limited species representation and the exclusion of Xenosauridae in the species samples used for this study.

Within Anguidae, Zheng et al. [30] placed Diploglossinae as a sister lineage to Anniellinae (Anguinae + Gerrhonotinae), supporting the monophyly of Anguidae. However, Pyron et al. [81] placed Anniellinae in a more distant position. This study did not include mitogenome data for Anniellinae, and increasing mitogenome data may help with classification within Anguidae. When the taxonomic units are sufficient, increasing the amount of genetic data is key to resolving differences.

### 3.3. Analysis of Divergence Time Estimation

Previous studies have estimated the origin of Squamata using different approaches, resulting in a range of ages due to variations in the number of taxa sampled, choice of molecular markers, and selection and number of fossil calibrations. Estimates had ranged from an average of 174.1 Mya for Squamata based on a ML tree using 4161 species and six fossil calibrations [83], to a root age of 281 Mya for Squamata based on the analysis of Albert et al.’s [68] of all mitogenomes from 37 squamate species and nine fossil calibrations. Earlier studies by Kumazawa et al. [69] and Vidal and Hedges [75] traced the origin of Squamata back to the Permian. However, our estimates of divergence dates suggested that the origin of crown-Squamata was in the late Triassic (206.05 Ma), which was consistent with recent studies of fossil evidence (206 Mya) [11], and findings on squamate anatomy (Late Triassic) [84]. And the major superfamilies and families within Squamata were largely consistent with previously published estimates.

In a series of studies employing mitochondrial genome datasets for phylogenetic analysis, researchers such as Kumazawa et al. [69] employed the Multidivtime and Bayesian autocorrelated clock methods, setting four calibration points on the outgroup. This approach resulted in divergence time estimates for 24 squamate species, with the crown Squamata estimated to be between 215 and 255 Mya. Okajima and Kumazawa [85] extended these methods by incorporating three fossil calibration points, leading to divergence times for 22 species, with the origin of crown-Squamata estimated at 240 Mya (220–260 Mya). On the other hand, Albert et al. [68] used r8s and Multidivtime methods, based on nine calibration points (five within the outgroup and four within Squamata), to estimate divergence times for 37 species, with the crown Squamata estimated to be older than 250 Mya. Their conclusion regarding root age was older than that inferred from our study, which employed the mcmctree method, utilizing the optimal topology derived from BI tree analysis and analyzing 213 samples. Our study employed seven fossil calibration points to determine divergence times of Squamata. Notably, their outgroup selection featured a broader range of calibrations, potentially influencing their results. When using the Multidivtime approach to derive divergence times, it allows for minimum and maximum constraints to be determined based on the fossil record. While minimum values are typically based on the earliest occurrences in the fossil record, maximum estimates are inherently more challenging and often involve subjective factors in selection, potentially resulting in inflated age estimates.

Mulcahy et al. [86] proposed that the origin of crown-Squamata occurred in 180 Mya, based on 25 nuclear loci from 64 squamate species and utilizing 14 fossil calibration points. Zheng and Wiens [87] estimated a significantly older age for the crown-Squamata (212.7 Mya), possibly reflecting a more reliable assessment given the employed methodology and shared fossil calibration points. The younger age estimated in Mulcahy et al. [86] may have been influenced by the artificially narrow age prior.

In Pyron’s [88] analysis using nuclear gene datasets, the median age of Squamata’s divergence was 189 Mya with four fossil calibration points, and 208 Mya with five calibration points. Wiens et al. [89] employed the semi-parametric penalized likelihood (PL) method with 11 fossil constraints, selecting the oldest known rhynchocephalian fossil to determine the MRCA of Squamata and Rhynchocephalia at 227 Mya, and resulting in a crown-group Squamata age of 178.7 Mya. Hugall et al. [90], also utilizing the PL method, opted for a maximum age of 450 Mya for the lungfish-tetrapod root, estimating the crown-group Squamata to be 171–201 Mya. The differences in estimated ages may be attributed to their chosen calibration schemes. The fossil calibration points selected in this study are mostly internal to Squamata. Recently, Zheng et al. [30] utilized the optimal tree inferred from a combined dataset and 13 fossil calibration points to estimate divergence times using TreePL v.1.0 software, and obtaining a date of 205.1 Mya for the crown-Squamata. Pyron [9] obtained an average estimate for the Squamata node at the Triassic-Jurassic boundary (186.8–199.6 Mya), based on morphological data and total evidence dating. Burbrink et al. [64] suggested the origin of crown-Squamata to be in the Early Jurassic (190 Ma), utilizing genomic data, for 289 samples, 75 families, and 26 fossils. An accurate origin time for Squamates remains elusive, with different evidence pointing in various directions. 

Similar to recent integrations of fossil, morphological, and molecular studies, many superfamilies subsequently originated during the Cretaceous period. The extensive continental splitting during the Cretaceous led to divergence among superfamilies. Groups originating in the Mesozoic rapidly diversified into all major subfamilies or genera in the Cenozoic, ultimately yielding 11,690 currently known extant species (perhaps underestimated). However, Kumazawa et al. [69] suggest that the extensive continental splitting during the Cretaceous is related to divergence at the level of lizard subfamilies or genera. The Cretaceous–Paleogene (K/Pg) boundary, as the fifth major extinction event, led to the elimination of nearly 75% of extant species, yet we did not find major lineages of Squamate originating during this period. Further information from the fossil record is needed to understand how the K/Pg boundary affected Squamate diversity. Previous studies have shown that a significant amount of missing data does not always pose a significant challenge to estimates of divergence times [30]. Perhaps finding reliable fossils can provide a more accurate assessment of the timing of Squamate origin and diversification.

### 3.4. Evolutionary History of Species in Anguinae

Molecular dating analysis indicated that the initial divergence of Anguinae occurred in approximately 38.70 Mya, which is older than the findings reported by Lavin et al. (about 26.47 Mya) [44] and by Gvoždík et al. (about 27.61 Mya). We supported the proposition that the Eocene epoch represented the earliest occurrence of Anguinae. This aligned with the timing of the smallest known Anguinae species discovered to date, *Headonhillia parva*, found in the Hampshire Basin [91]. Although Anguinae did not display a high species richness compared to other lineages, their extensive distribution can be attributed to their timely appearance in suitable habitats. During the Oligocene (ca. 33.9 mya), the global climate cooled, leading to fragmentation of forest habitats and expansion of grasslands. Taking advantage of their snake-like bodies capable of maneuvering through grass, they exploited this expanding ecological niche, facilitating speciation and clade radiation. The MRCA of *Anguis* and *Pseudopus*, as well as the divergence of the genus *Dopasia*, occurred shortly after the Oligocene epoch. The subfamily Anguinae originated from an ancestor within Anguidae in Europe. Currently, *Pseudopus* and *Anguis* formed an important sister group within the European lineage, and both were monophyletic. The MRCA of this group predated the estimate proposed by Lavin et al. [44] using BEAST v. 1.8.2, as well as the estimate by Gvoždík et al. [92]. In the genus *Anguis*, the morphology of *Anguis cephallonica* was distinct and differs significantly from that of the other species. This distinction allowed for the division of *Anguis* into two groups: *A. cephallonica* and the *A. fragilis* species complex. The *A. fragilis* species complex was previously divided into three species, the widespread western *A. fragilis*, the widespread eastern *A. colchica,* and the southwestern Balkan endemic *A. graeca*. Later, *A. veronensis* was added to the complex. However, based on the phylogenetic tree, the relationship between *A. veronensis* and *A. cephallonica* appeared to be closer. Research had suggested that the speciation of *A. veronensis* is associated with the Messinian event [92,93,94]. 

In the case of the diversification of *P. apodus* into three subspecies, the timeframe was earlier than the research conducted by Gvoždík et al. [92]. The second and third major lineages were composed of Asian (*Dopasia*) and North American species (*Ophisaurus*), respectively, and they exhibited a sister relationship as continental lineages. Due to the lack of mitogenomes from species in the genus *Ophisaurus*, it remains unclear when the North American branch represented by the genus *Ophisaurus* diverged from the Asian lineage. Within the Asian lineage, *D. gracilis* and *D*. *sokolovi* formed a clade that was sister to another clade consisting of *D*. *hati* distributed from eastern Vietnam to Taiwan and *D*. *hainanensis* originating from Hainan Island, China [95]. This finding is consistent with previous studies [44,96].

### 3.5. Selective Pressure within Anguimorpha

Factors that have an impact on the morphology, behaviour, and physiology of a species, or that drive speciation and evolutionary innovation, include favorable genetic and genomic mutations. However, beneficial mutations are indeed rare at the molecular level, as positive selection only impacts a limited number of amino acid sites during a relatively short period of evolution, and these positively selected sites often succumb to subsequent negative selection processes. Limb loss has evolved independently at least 26 times in Squamata [89,97], and the shift in body morphology from lizards to snakes is one of the most significant transitions in reptile evolution [98]. Numerous lineages of squamates have given rise to limb-reduced and elongated (serpentiform) species, that demonstrates the evolutionary success of this modification from the ancestral lizard *Bauplan* [99].

The movement patterns of limbless lizard species exhibit similarities to the undulating and serpentine locomotion observed in snakes, relying predominantly on a push-slide mode of propulsion [100]. In this study, selection pressure analyses were conducted on limbless lizards within the Anguimorpha clade using the branch site model and the clade branch model. The consistent results of two analyses indicated that the evolution of limb loss in these lizards was influenced by Darwinian natural selection. Interestingly, the branch-site models identified nine out of the ten positively selected sites on mitochondrial complex I, suggesting a stronger positive selection signal on mitochondrial complex I compared to other mitochondrial complexes. Complex I, the largest protein in the mitochondrial respiratory chain, consists of 41 subunits and catalyzes the transfer of electrons from NADH to ubiquinone, generating over one-third of the energy produced in the mitochondria [101,102,103]. Among these subunits, *ND1*-*ND4*, *ND4L*, *ND5*, and *ND6*, that are encoded by mtDNA, account for a total of seven subunits [104]. The hydrophobic portion of mitochondrial respiratory complex I is composed of various proteins. One component, NuoL (equivalent to ND5), forms a piston arm that interacts with three proton pumps: NuoL (ND5), NuoM (ND4), and NuoN (ND2) [105]. These structures place mitochondria at the center of metabolism and bioenergetic conversion. Loss or mutation of the aforementioned subunits can lead to changes in the protein structure composing mitochondria or the protein transport mechanisms within them. While limbless lizards have modified their locomotion compared to their limbed counterparts, our study discovered that limbless lizards experienced positive selection. Additionally, their mitochondrial codons were reorganized to fulfil the amino acid requirements of proteins. *Dibamus bourreti*, a species where locomotion is supported by the combined movement of the remaining limbs and body swing, underwent selection in the *ATP6* gene as compared to species with limbs [106]. This implies that the mitogenome plays a critical role in meeting energy demands following locomotion pattern alterations in both limbed and limbless lizards.

The loss of limbs is typically associated with various morphological changes, such as elongation of the body, asymmetry of internal organs, and the development of specialized structures in the epidermis of the skin [99,100]. Recent molecular evidence has emerged, providing a connection between the loss of limbs and changes in locomotion patterns [107]. The results of this study supported this idea and aligned with Dollo’s Law of irreversible evolution [108,109], a widely accepted hypothesis stating that once complex structures are lost, they cannot be reacquired, even in isolated traits. It is considered unlikely for the same complex structures to evolve again from scratch. However, if the underlying genetic framework responsible for the lost trait remains intact, there is a possibility that reactivated genes could lead to its restoration. Nonetheless, in the case of limbless lizard populations, the re-emergence of limbs has not occurred. This could be attributed to their preference for a fossorial lifestyle or their need to navigate sandy or densely grassy habitats [58,60,110], where limbs would hinder their movements and impose significant energy costs.

## 4. Materials and Methods

### 4.1. Sample Collection and DNA Extraction

We acquired the mitogenomes of two species from the Anguidae family, namely *Pseudopus apodus* from Dedoplistskaro, Georgia (41°26′ N, 46°06′ E), and *Dopasia gracilis* from Baoshan, Yunnan (25°00′ N, 99°04′ E). Additionally, samples from three other families were used for mitogenome sequencing and comparative analysis: Scincidae: *Tiliqua gigas gigas* from Halmahera, Indonesia (23°11′ N, 113°18′ E); *Chalcides ocellatus* from Boulemane, Morocco (32°54′ N, 3°59′ E); *Cyclodomorphus gerrardii* from New South Wales, Australia (29°42′ N, 152°32′ E) and *Plestiodon quadrilineatus* from Guilin, GuangXi (25°48′ N, 110°13′ E); Gekkonidae: *Gekko chinensis* from Guilin, Guangxi (25°15′ N, 110°21′ E) and *Gekko japonicus* from Jinhua, Zhejiang (28°52′ N, 120°04′ E). All specimens were collected during 2010 and were stored at the Animal Specimen Museum of Zhejiang Normal University to promote popular scientific knowledge (ZJNU-20100711-DWSX001, CSX002, ZGBH004, DYBH005, LSSLZ007, TSSLZ008, SXSLZ009, YBSLZ010). A 2 mm tail sample was taken from each species, and DNA was extracted using an Ezup Column Animal Genomic DNA Purification Kit (Sangon Biotech Company, Shanghai, China). The DNA information was obtained following the instructions provided by the manufacturer.

### 4.2. PCR Amplification and Sequence Capture

Two mitogenomes (*Pseudopus apodus* and *Dopasia gracilis*) were obtained by Sanger sequencing. TaKaRa *rTaq* and TaKaRa *LA-Taq* DNA polymerase were used to perform short fragment amplification (<3000 bp) and long fragment amplification (>3000 bp), respectively. First, several partial segments were amplified using common primers of lizards [111]. Second, species-specific primers were designed to use Primer Premier 5.0 (Primer Biosoft International, San Francisco, CA, USA) [112] to link the gaps where some fragments (reads) were not assembled by common primers for lizard mitogenomes, as described by Zhang et al. [113]. The PCR products were checked by electrophoresis in a 1% agarose gel, and sequencing of the PCR products was carried out directly by Sangon Biotech Company (Shanghai, China).

DNA extracts from the other six species with concentrations exceeding 25 µg/mL were sent to BGI Tech Inc. (Shenzhen, China) for next-generation sequencing (NGS). Genomic DNA was sequenced on the Illumina HiSeq 2000 platform with 150 bp paired-end reads. After quality assessment of the raw sequencing data using fastQC, clean data were used for genome assembly.

### 4.3. Mitogenome Annotation and Sequence Analyses

The fragments were assembled into a mitogenome and aligned using SeqMan in the DNASTAR Package V.7.1 [114], which showed that there were at least 50 bp repeats and a single peak pattern. NGS were assembled in NOVOPlasty v.4.2 [115], GetOrganelle v.1.7.1 [116]. Localization of tRNA genes of the mitogenome was annotated via the MITOS web server (http://mitos2.bioinf.uni-leipzig.de/index.py, accessed on 9 May 2023) [117]. With reference to the annotated lizard genomes from the GenBank online database, 13 PCGs in six lizards included in this study were manually adjusted. These codons were then tested in MEGA7.0 [118] for successful translation into amino acids according to the vertebrate mitogenome genetic code as well as codon usage to determine the location of the 13 PCGs for the sequence. In addition, analysis of mitogenome structure was performed using PhyloSuite v1.2.3 [119], including assessment of AT content and relative synonymous codon usage (RSCU) in PCGs. The GC and AT skew was obtained through the following calculation [120]: AT skew = (A − T) ÷ (A + T), GC skew = (G − C) ÷ (G + C). Maps of complete mitogenomes were generated using the CG View online server V 1.0 (https://cgview.ca/, accessed on 9 May 2023) [121].

### 4.4. Phylogenetic Analyses

In this study, we utilized the 13 PCGs and two rRNAs (12S rRNA and 16S rRNA) from 213 mitogenomes to construct phylogenetic trees. This included eight mitochondrial sequences obtained from our present study and 205 additional mitogenomes retrieved from the NCBI database. The samples covered representatives from three major taxa of Squamata: lizards (145 sequences) [22,67,68,69,70,79,85,92,96,106,122,123,124,125,126,127,128,129,130,131,132,133,134,135,136,137,138,139,140,141,142,143,144,145,146,147,148,149,150,151,152,153,154,155,156,157,158,159,160,161,162,163,164,165,166,167,168,169,170,171,172,173,174], amphisbaenians (eight sequences) [68,69,175], and snakes (51 sequences) [77,141,176,177,178,179,180,181,182,183,184,185,186,187,188,189,190,191,192,193] (Table S6), ensuring a comprehensive representation for our phylogenetic analysis. Based on the research conducted by Zheng et al. [30], seven primitive species including *Sphenodon punctatus*, *Alligator mississippiensis*, *Crocodylus porosus*, *Dromaius novaehollandiae*, *Gallus gallus*, *Chelydra serpentina,* and *Podocnemis expansa* were employed as outgroups [194,195,196] (Table S6).

To import all mitogenome sequences, including the GenBank accession numbers and GB format, for gene extraction into PhyloSuite v1.2.3 [119], the following steps were carried out: First, perform nucleotide sequence alignment of the 13 PCGs and two rRNAs using MAFFT V.7.475 [197]; then, conduct sequence conservation analysis using Gblock 0.91b [198]; and finally, concatenate the sequences into a single sequence using the Concatenate Sequence module within PhyloSuite v1.2.3 [119].

The third codon was detected to be saturated using DAMBE [199], so the phylogenetic tree was constructed using the first and the second codons. To enhance the reliability of the constructed phylogenetic tree, the best partitions and substitution models (Table S7) for tree construction were obtained through screening using PartitionFinder 2.2.1 [200] based on the Bayesian information criterion (BIC). The partitioning results were used to perform BI analysis in MrBayes version 3.2 [201]. The BI analysis was set to run for 10 million generations, with trees being sampled every 1000 generations. It was configured to stop when the standard deviation of the separation frequency was less than 0.01. The initial 25% of the generations were discarded as burn-in. Posterior probabilities were calculated for Bayesian support. ML analysis was performed in RAxML v.8.2, and a total of 1000 runs were performed with the bootstrap value set to 100 [202].

### 4.5. Divergence Dating Estimation

Fossil evidence is one of the main research methods used to estimate the origins and divergence times of species, and it is also a direct method. Based on fossil evidence and references to previous studies, we selected six calibration points within Squamata, as well as one in an outgroup: (1) 238 Mya was chosen as the minimum age for Rhynchocephalia–Squamata split [203], and 252 Mya was taken as the maximum [204]. (2) *Titanoboa* was placed in the Boinae on the basis of derived characters of the vertebrae. The fossils came from the La Puente Pit in the Cerrejón Coal Mine, palynological zone Cu-02, dated to the middle-late Paleocene [205], so we set 58 Mya as the hard minimum and 66 Mya as the soft maximum. (3) *Protodraco monocoli* was chosen as the calibration point for Agamidae [206]. (4) *Anniealexandria* from the Early Eocene was discovered as a bipedid, making it the oldest (indeed, the only) fossil representative of its family [37]. (5) *Primaderma* was placed as the calibration point for the node *Heloderma suspectum*, representing a helodermatid-like lizard [207]. (6) The *Gerrhonotus* fossil calibration point was positioned at the MRCA of Gerrhonotinae and Anguinae [208,209]. (7) According to fossil evidence of *Anguis rarus* and *Pseudopus ahnikoviensis* [46,54], the minimum age for *Anguis rarus* and *Pseudopus ahnikoviensis* was set at 18.4 Mya, and the maximum age at 30 Mya, placing them at the MRCA of *Anguis* and *Pseudopus*. For more details, please refer to Table 5.

Based on the constructed BI tree, the MCMCTree program from the PAML v.4.8 Package [210] was used to explore the divergence times of major branches within Squamate. The analysis involved several steps. First, the Baseml subroutine in the PAML v.4.8 software was utilized to calculate the nucleotide substitution rates (regene gamma). For this step, sequence-related phy files, tree files with rooted topology including branch lengths and ctl configuration files, needed to be prepared. Next, the branch lengths were computed. The phy file was the same as in the previous step, while the tree file incorporated the substitution rates obtained from the first step. The values of burnin, sampfreq, and nsample were adjusted based on the size of the dataset. Once the branch-length file was obtained, the analysis of divergence times was performed. Finally, the tree with divergence times was obtained, and visualization and inspection were conducted using the FigTree v1.4.0 program [211]. Tracer v.1.7.1 software [212] was used to analyze the resulting mcmc.txt file generated by the analysis and to check if the effective sample size (ESS) values were higher than 200, indicating convergence.

### 4.6. Detecting Selective Pressure

Natural selection was recognized as one of the five genetic forces (mutation, recombination, selection, gene flow, genetic drift), and it exerted a significant influence on codon usage bias within the mitogenomes of reptiles [213]. The analysis of selective pressures was an essential and integral component in the field of evolutionary analysis. Nucleotide variations that do not result in changes to the amino acid sequence were referred to as synonymous mutations, whereas those that led to changes in the amino acid sequence were known as nonsynonymous mutations. Using the synonymous substitution rate as a criterion, it was possible to infer whether the retention of nonsynonymous mutations is supported or impeded by natural selection. The ratios of ω < 1, ω = 1, and ω > 1 correspond to purifying selection, neutral selection, and positive selection, respectively [210].

EasyCodeML 1.41 [214], as an alternative to Codeml, implements visualized operations and can be used for detecting selection in molecular evolutionary analysis. Alignment was performed on the 13 PCGs from a total of 23 sequences in Anguimorpha, resulting in 11,268 nucleotide sites (excluding start and stop codons) and 3756 amino acid sites. Analysis of selection pressure was conducted on these sites. This study utilized two different models from the EasyCodeML module [214]. The branch-site model posits that variations in selective pressures exist among different amino acid sites as well as among different lineages. It took into account the differences in ω values not only between sites but also between lineages. Due to disparities in movement and energy distribution between limbless lizards and fully limbed lizards, the limbless lizards from Anguinae were selected as the foreground branch, whereas the remaining limbed lizards were considered the background branch. The commonly used pair of models is Model A vs. Model A null. Determination of positive selection in the foreground branch was based on the *p* value. If the *p* value from the likelihood ratio test (LRT) was less than 0.05, it indicated the presence of positive selection in that lineage. Conversely, if *p* > 0.05, there was no significant difference between the foreground and background branches. Additionally, the Bayesian empirical Bayes (BEB) method was employed to calculate the posterior probability of amino acid sites in each positively selected lineage. Sites with BEB > 0.5 were considered potential positive selection sites, whereas sites with BEB > 0.95 were considered to be potentially stronger positive selection sites. The clade model has been capable of detecting positive selection sites in multiple branches of evolution [210]. In this study, only the limbless lizards in Anguimorpha were selected as the foreground branch. UniProt [215] and SWISS-MODEL Workspace [216] were used to gather information on the structure and function of positively selected genes and to construct the corresponding protein three-dimensional (3D) structure, respectively.

## 5. Conclusions

The results of the BI tree and ML tree constructed used 213 complete mitochondrial sequences of 13 PCGs (the first and the second codons) and two rRNAs from Squamata species including nine outgroups closely related to Squamata. Both trees recovered the monophyly of Dibamia, Gekkota, Anguimorpha, Amphisbaenia, and Serpentes. Gekkota was placed at the root of the Squamata tree. Additionally, it aligns with the findings from the majority of recent molecular evidence, confirming the compatibility with the majority of topological structures. However, the support values for both trees at the deep nodes of Squamata are low, reflecting the instability of the relationships among the lineages. Here, our estimates of divergence dates suggested that the origin of crown-Squamata was in the Late Triassic (206.05 Mya). With Anguimorpha, compared to limbed lizards, there were 10 positively selected sites in the mitochondrial genes of limbless lizards. The 10 positively selected sites were as follows: Cytb protein (at sites 183 and 187), ND2 protein (at sites 90, 155, and 198), ND3 protein (at site 21), ND5 protein (at sites 12 and 267), and two sites (72 and 119) in ND6 protein. This suggests that limbless lizards are undergoing active selection in their mitochondrial genes to balance the energy-allocation differences caused by their mode of locomotion.

## Figures and Tables

**Table 1:** Composition of the mitogenomes of the eight species in this study, with 13 protein-coding genes located on the heavy strand.

Species	Whole Genome				PCGs			
	Length (bp)	A+T%	AT-K	GC-K	Length (bp)	A+T%	AT-K	GC-K
*Dopasia gracilis*	15,855	54.3	0.124	−0.344	11,379	53.8	0.064	−0.386
*Pseudopus apodus*	16,274	55.2	0.112	−0.346	11,388	54.7	0.053	−0.380
*Cyclodomorphus gerrardii*	16,093	59.3	0.086	−0.335	11,373	59.5	0.019	−0.386
*Chalcides ocellatus*	16,563	57.9	0.143	−0.330	11,388	57.8	0.099	−0.375
*Tiliqua gigas gigas*	16,957	57.7	0.078	−0.317	11,367	57.9	0.021	−0.380
*Plestiodon quadrilineatus*	17,391	56.5	0.120	−0.322	11,382	56.1	0.076	−0.357
*Gekko chinensis*	17,657	60.9	0.100	−0.315	11,298	60.6	0.062	−0.361
*Gekko japonicus*	17,707	56.6	0.113	−0.322	11,319	55.8	0.075	−0.368

**Table 2:** Divergence times of nodes and clades within Squamata based on the mt genomes. All estimates are expressed as million years ago (Mya). The 95% highest posterior densities (HPD) are shown in the right column of the table. “&” represents the relationship between two branches.

Nodes/Clades	Mean Divergence Time (Mya)	95% HPD Range (Mya)
Squamata root	206.05	182.08~229.55
Gekkota	155.31	123.30~186.71
(Scincidae + Dibamia) & (Cordylidae + Xantusiidae)	124.87	86.53~194.85
Scincidae	75.79	58.95~93.74
Anguimorpha	111.91	102.35~126.18
Pleurodonta	88.86	70.27~102.38
Acrodonta	117.89	103.23~137.10
Serpentes	135.31	115.23~154.91
Acrodonta & Serpentes	162.67	141.23~183.67
Anguimorpha & Pleurodonta + (Acrodonta + Serpentes)	184.12	160.76~206.52
Amphisbaenia	149.39	121.61~176.25
Lacertoidea (Gymnophthalmidae, Lacertidae, Amphisbaenia)	167.20	151.53~200.53
*Dopasia* & (*Pseudopus* + *Anguis*)	38.70	31.18~46.33
*Pseudopus* & *Anguis*	27.26	22.85~30.51
(*Dopasia gracilis* + *Dopasia sokolovi*) & (*Dopasia hati* + *Dopasia hainanensis*)	30.26	21.94~38.83

**Table 3:** Parameters and results analysed by the Branch-site model in Anguimorpha. (*p* < 0.05 indicates a significant difference.)

Foreground Branch	Model	np	Ln L	Estimates of Parameters					Model Compared	LRT *p*-Value	Positive Sites
*Anguis*	Model A	49	−81,825.55313	Site class	0	1	2a	2b	Model A vs. Model A null	0.00000	1151 E 0.989 *,1216 T 0.972 *, 1259 N 0.959 *,1688 A 0.987 *, 2249 T 0.973 *,2504 F 0.983 *, 3138 S 0.956 *,3187 T 0.991 **, 3464 A 0.979 *,3468 G 0.984 *
*Dopasia*				f	0.83441	0.10720	0.05174	0.00665			
*Pseudopus*				ω_0_	0.04478	1.00000	0.04478	1.00000			
	Model A null	48	−81,845.54159	ω_1_	0.04478	1.00000	7.91959	7.91959			
				1							Not Allowed

Note: * and ** indicate BEB values > 0.95 and > 0.99, respectively.

**Table 4:** The features and description of the positive selection sites detected in the mitochondrial PCGs of limbless lizards in Anguimorpha.

Genes	Positive Selection Sites	Amino Acids		BEB Value	Feature Key *	Description
		Foreground	Background			
ND2	90	E	S\T	0.989 *	Transmembrane	Helical
	155	T\M	L	0.972 *	Transmembrane	Helical
	198	N	T\P	0.959 *	/	/
ND3	21	A	S	0.987 *	Transmembrane	Helical
ND5	12	T\A	L	0.973 *	Transmembrane	Helical
	267	F\S	H	0.983 *	Transmembrane	Helical
ND6	72	S	A\S	0.956 *	Transmembrane	Helical
	119	T	G\D	0.991 **	Transmembrane	Helical
CYTB	183	A	L	0.979 *	Transmembrane	Helical
	187	G	I	0.984 *	Transmembrane	Helical

Note: * and ** indicate BEB values > 0.95 and > 0.99, respectively.

**Table 5:** Fossil calibration points used for estimating dates of divergence (nodes in Figure 6).

Node Figure 6	Minimum Age of Fossil Constraint (Mya)	Maximum Age of Fossil Constraint (Mya)	Fossil Calibration	Age (Period/Stage)	References
1	238	252	Rhynchocephalia-Squamata	Middle Triassic	[203,204]
2	58.00	66.00	*Titanoboa cerrejonensis*	Paleocene	[205]
3	93.30	99.60	*Protodraco monocoli*	Late Cretaceous	[206]
4	47.80	55.80	*Anniealexandria*	Early Eocene	[37]
5	99.60	102.70	*Primaderma*	Early Cretaceous	[207]
6	48.60	57.00	*Gerrhonotus*	Lower Eocene	[208,209]
7	18.40	30.00	*Anguis rarus*, *Pseudopus ahnikoviensis*	Early Neogene	[46,54]

## Data Availability

Data to support this study are available from the National Center for Biotechnology Information (https://www.ncbi.nlm.nih.gov, accessed on 26 August 2023). The registration numbers are PP003927-PP003930 and PP571905-PP571908.

## Supplementary Materials

The following supporting information can be downloaded at: https://www.mdpi.com/article/10.3390/ijms25158464/s1.
