# Supplementary figures and images for: The Phylogenetic Relationships of Major Lizard Families Using Mitochondrial Genomes and Selection Pressure Analyses in Anguimorpha
Source: Int J Mol Sci. 2024 Aug 2;25(15):8464. doi: 10.3390/ijms25158464 (PMC11312734; doi:10.3390/ijms25158464)

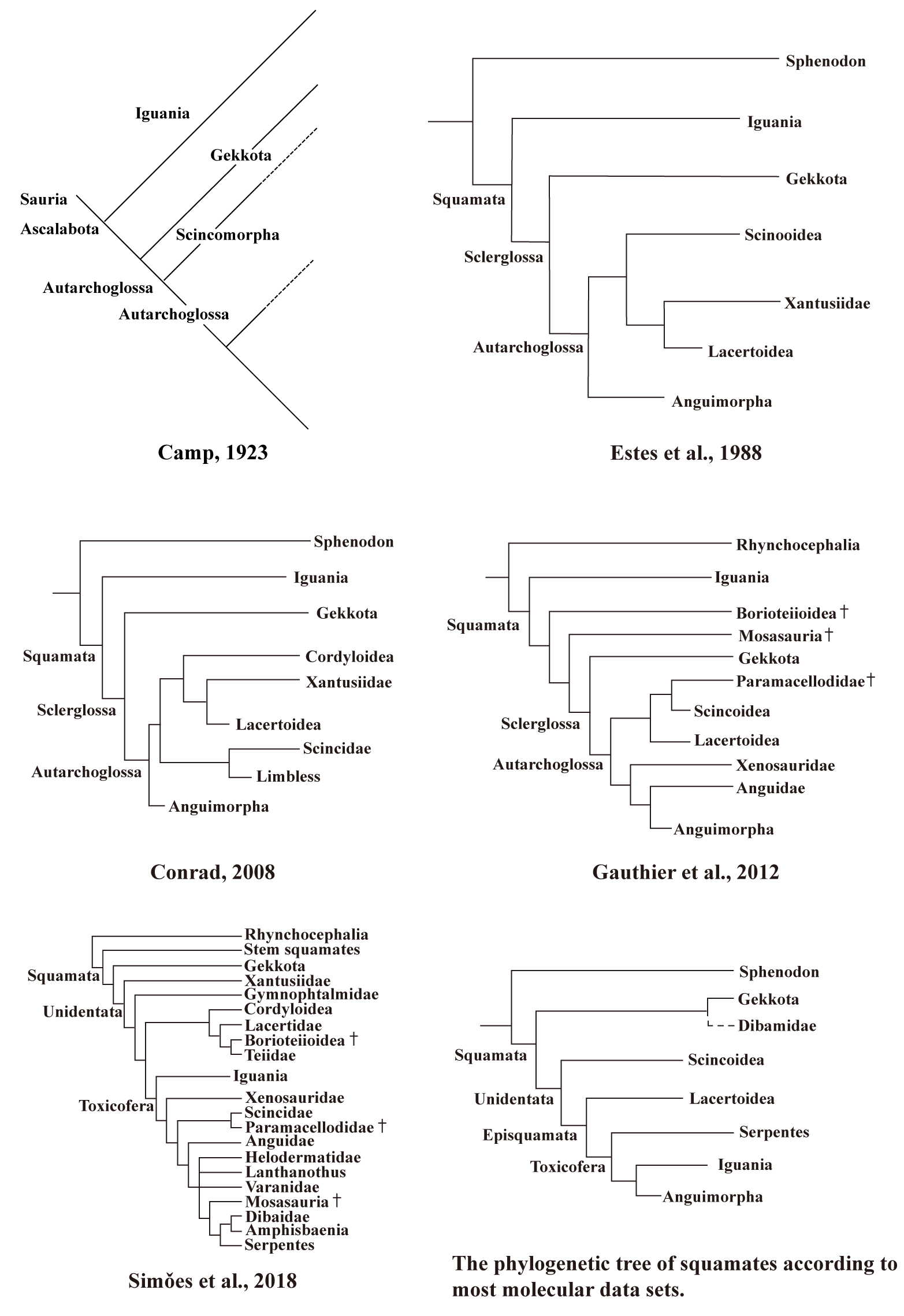

Supplement: Supplementary file 1 [file ijms-25-08464-s001.zip › Figure S1.Squamate tree hypothesis .png]
